# Molecular Attributes Associated With Refolding of Inclusion Body Proteins Using the Freeze–Thaw Method

**DOI:** 10.3389/fmicb.2021.618559

**Published:** 2021-04-20

**Authors:** Priyank Singhvi, Juhi Verma, Neha Panwar, Tabiya Qayoom Wani, Akansha Singh, Md. Qudratullah, Arnab Chakraborty, Ankit Saneja, Debi P. Sarkar, Amulya K. Panda

**Affiliations:** ^1^Product Development Cell, National Institute of Immunology, New Delhi, India; ^2^Department of Biochemistry, University of Delhi, New Delhi, India

**Keywords:** inclusion bodies, mild solubilization method, freeze–thaw method, protein refolding, human growth hormone, protein aggregates, L-asparaginase

## Abstract

Understanding the structure–function of inclusion bodies (IBs) in the last two decades has led to the development of several mild solubilization buffers for the improved recovery of bioactive proteins. The recently developed freeze–thaw-based inclusion body protein solubilization method has received a great deal of attention due to its simplicity and cost-effectiveness. The present report investigates the reproducibility, efficiency, and plausible mechanism of the freeze–thaw-based IB solubilization. The percentage recovery of functionally active protein species of human growth hormone (hGH) and L-asparaginase from their IBs in *Escherichia coli* and the quality attributes associated with the freeze–thaw-based solubilization method were analyzed in detail. The overall yield of the purified hGH and L-asparaginase protein was found to be around 14 and 25%, respectively. Both purified proteins had functionally active species lower than that observed with commercial proteins. Biophysical and biochemical analyses revealed that the formation of soluble aggregates was a major limitation in the case of tough IB protein like hGH. On the other hand, the destabilization of soft IB protein like L-asparaginase led to the poor recovery of functionally active protein species. Our study provides insight into the advantages, disadvantages, and molecular–structural information associated with the freeze–thaw-based solubilization method.

## Introduction

Since the inception of recombinant DNA technology, *Escherichia coli* has been most widely used as a host to produce recombinant proteins whose bioactivity is not dependent on posttranslational modifications. Almost 80% of overexpressed recombinant proteins in *E. coli* result in the formation of protein aggregates known as inclusion bodies (IBs) (Jürgen et al., 2010). IBs, in general, are highly dense particles having low aqueous solubility. Solubilization of the inclusion body proteins and refolding of the solubilized protein are the major determining steps for the efficient recovery of bioactive proteins (Singh and Panda, 2005).

Conventional methods for inclusion body solubilization involve the usage of a high concentration of denaturing agents such as urea or guanidine hydrochloride along with a reducing agent (Fischer et al., 1993; Rudolph and Lilie, 1996). Although these methods solubilize almost all of the (IBs), the recovery of bioactive protein is very low due to the formation of aggregates during refolding (Datar et al., 1993; Panda, 2003). Concomitantly, the presence of a native-like secondary structure of the protein trapped and the confirmation of biological activity in IBs led to the development of several mild solubilization buffers in the past two decades (Ventura and Villaverde, 2006; Singhvi et al., 2020). Mild denaturing buffers are strong enough to solubilize bacterial (IBs) but mild enough to protect the existing native-like secondary structure of the protein in IBs. These buffers are based on alkaline and acidic pH, high hydrostatic pressure, organic solvents, and freeze–thaw in the presence of a low concentration of chaotrope. In the past two decades, the buffers have consistently demonstrated improved recovery of bioactive protein from bacterial (IBs) (Singh et al., 2015).

Out of several mild solubilization methods developed in the past, the recently developed freeze–thaw-based inclusion body protein solubilization method has received a great deal of attention due to its simplicity and cost-effectiveness. The method involves rapidly freezing the IB suspension in the presence of 2 M urea present in a buffer (pH 7–9) at −20°C overnight and quickly thawing at room temperature (Qi et al., 2015). The solubilization potential of the freeze–thaw-based method has been reported with different inclusion body proteins (Maruthamuthu et al., 2017; Schultz-Johansen et al., 2018; Sadeghian-Rizi et al., 2019). However, rapid freezing and quick thawing of the protein is reported to promote aggregation and instability and thereby reduce the functionally active protein species (Pikal-Cleland et al., 2000; Bhatnagar et al., 2007; Desu and Narishetty, 2013). Therefore, it was of interest to investigate the reproducibility, efficiency, and plausible mechanism of IB solubilization based on a previously reported freezing and thawing method.

In this study, we have taken tough (IBs) like human growth hormone (hGH) and soft (IBs) like L-asparaginase (Upadhyay et al., 2014, 2016). hGH is a single-chain polypeptide of 191 amino acids having two disulfide bonds and is involved in cellular growth and regeneration (Gunawardane et al., 2000). L-Asparaginase is a homotetrameric protein where each monomer is made up of 348 amino acids with a molecular mass ∼37 kDa and has one disulfide bond (UniProt ID: P00805). Previously reported mild denaturing buffers have shown overall yield for both bacterial (IBs) to be around 50%. However, the overall yield and recovery of functionally active protein from both (IBs) using the freeze–thaw method have not been analyzed and reported yet. In addition, the molecular–structural attributes associated with the freeze–thaw-based solubilization method were also investigated in solubilized and refolded states of proteins using various biophysical and biochemical tools. This was achieved by analyzing the secondary and tertiary structures of the proteins in the solubilized state, the effect of refolding concentration and time duration on the formation of soluble aggregates, and the stability of the protein with respect to time. Finally, we discuss the advantages, disadvantages, and plausible mechanism associated with the freeze–thaw-based solubilization method for bacterial (IBs).

## Materials and Methods

### Chemicals and Reagents

Culture media components such as tryptone extract and Bacto yeast extract were purchased from Difco Laboratories, India. Phenylmethylsulfonyl fluoride (PMSF), isopropyl β-D-1-thiogalactopyranoside (IPTG), acrylamide, bis-acrylamide, Tris–HCl, and L-asparagine were from Amresco, United States. Sodium dodecyl sulfate (SDS), ammonium persulfate (APS), dithiothreitol (DTT), urea, Nessler’s reagent, and trichloroacetic acid (TCA) were from Sigma-Aldrich, United States. 2,2,2-Trifluoroethanol was from SRL, India. Tetramethyl ethylenediamine (TEMED), ethylenediaminetetraacetic acid (EDTA), and bromophenol blue were from Bio-Rad, United States. Coomassie Brilliant Blue R-250 and ampicillin were from USB Corporation, United States. Glacial acetic acid and methanol were from Merck’s EMPARTA^®^. Ethanol, *n*-propanol, and glycerol were of analytical grade and were from Spectrochem, India. DEAE-Sepharose Fast Flow media was purchased from GE Healthcare, United Kingdom. BCA assay kit, SDS-PAGE prestained molecular weight marker, and commercial r-hGH were from Thermo Fisher Scientific, United States. Commercial L-asparaginase 5,000 IU/ml, Bionase^®^ 5K, was purchased from Zydus Cadila, India. RPMI, horse serum (HS), and fetal bovine serum (FBS) were from Gibco BRL, United States. All the other chemicals were of analytical grade.

### Expression of Recombinant hGH and L-Asparaginase

Primary cultures of *E. coli* expressing hGH and L-asparaginase were grown overnight in modified Luria–Bertani (LB) media (5 g/L glucose, 5 g/L yeast extract, 10 g/L tryptone, and 10 g/L NaCl) from the glycerol stock. Antibiotics such as ampicillin (Amp) and kanamycin (Kan) were added into the primary culture of hGH, whereas only ampicillin was used in that of L-asparaginase. The final concentration of Kan and Amp used was 50 and 100 μg/ml, respectively. A medium containing suitable antibiotic(s) was inoculated with 1 ml glycerol stock of *E. coli* and incubated overnight at 37°C and 200 rpm in an orbital shaker (Kühner shaker, Switzerland).

Primary cultures of *E. coli* cells [1–2% (v/v) final concentration] grown overnight were inoculated into 1 L of LB media containing ampicillin (100 μg/ml) and kanamycin (50 μg/ml) antibiotics and were incubated at 37°C and 200 rpm. The growth of *E. coli* broth was monitored by measuring absorbance at 600 nm in a UV–Vis spectrophotometer (Amersham Biosciences, United Kingdom). At an OD of 0.6, the culture was induced by adding IPTG as an inducer wherein the final concentration of IPTG was 1 mM. The bacterial cultures were harvested 4 h postinduction by centrifugation at 4°C at 4,000 rpm for 15 min (Sorvall RC6+, United States) and stored at −20°C for further use.

### Isolation and Purification of hGH and L-Asparaginase IBs

*E. coli* cell pellets from a 1-L shaker flask culture were resuspended in 50 ml lysis buffer (50 mM Tris–HCl, 5 mM EDTA, and 1 mM PMSF) and homogenized. The homogeneous suspensions were further sonicated on ice at an amplitude of 50% for 10 cycles with 1 min delay time wherein each cycle of 1 min includes alternate pulses for 1 s on and off (Q700 sonicator, Qsonica, United States). Lysed bacterial cells were centrifuged (12,000 rpm, 4°C, 30 min, Eppendorf Centrifuge 5810 R) and inclusion bodies (IBs) were obtained in the pellet. The IBs were washed by resuspending the pellet in 50 ml of Milli-Q (MQ) water and centrifuging (12,000 rpm, 4°C, 30 min, Eppendorf 5810 R, Germany). The washed IBs were finally resuspended in 2 ml of MQ water.

### Solubilization and Refolding of Recombinant hGH and L-Asparaginase

Two milliliters of the IBs obtained from the 1-L culture were solubilized in 18 ml of 2 M urea with 1 mM DTT and rapidly frozen at −20°C overnight. The frozen sample was quickly thawed at room temperature and then centrifuged (12,000 rpm, 4°C, 30 min, Eppendorf Centrifuge 5810 R, Germany). The hGH and L-asparaginase proteins in the supernatant were diluted in a pulsatile manner in 180 ml of the respective refolding buffers [50 mM Tris–HCl, pH 8.5 for solubilized hGH and 50 mM Tris–HCl, 10% (v/v) glycerol, pH 8.5 for solubilized L-asparaginase]. The refolded samples were then centrifuged (12,000 rpm, 4°C, 30 min) to obtain the soluble and refolded proteins in the supernatant. The supernatants were then used for protein purification through liquid chromatography.

### Purification of the Refolded hGH

Refolded protein was purified using anion exchange and size exclusion chromatography. Eight milliliters of DEAE-Sepharose Fast Flow media, anion exchange, was manually packed in a column (Pharmacia, Sweden) connected with ÄKTA pure (GE Healthcare, United Kingdom). The column was washed with 100 ml of MQ water and equilibrated with 50 ml of equilibration buffer (50 mM Tris–HCl, pH 8.5). Two hundred milliliters of refolded protein was loaded onto the DEAE-Sepharose column and washed with 30 ml of equilibration buffer. Elution was carried out using the NaCl gradient (0–500 mM NaCl) for 100 ml. Washing, equilibration, protein loading, and elution were performed at a flow rate of 2 ml/min. Eluates were collected in 5 ml fractions and the presence of protein was further confirmed by SDS-PAGE.

Eluates containing protein were pooled and concentrated to 3 ml using a concentrator (Amicon^®^ Ultra-15 Centrifugal Filter Units, Merck Millipore, United States) with the membrane of 3 kDa molecular weight cutoff. The concentrated sample was centrifuged (12,000 rpm, 4°C, 10 min) before loading onto the size exclusion chromatographic column attached with ÄKTA pure (GE Healthcare, United Kingdom). The size exclusion column Superdex 75 PG (GE Healthcare, United Kingdom) was washed with 120 ml of MQ water and equilibrated with 120 ml of 20 mM Tris–HCl, pH 8.5. Three milliliters of the concentrated sample was loaded onto the equilibrated column and eluted in 120 ml of 20 mM Tris–HCl. Washing, equilibration, and elution were performed at a flow rate of 0.5 ml/min. The quality of the purified protein was checked on SDS-PAGE.

### Purification of the Refolded L-Asparaginase

Refolded L-asparaginase was purified using DEAE-Sepharose anion exchange matrix packed in XK 16 column (GE Healthcare, Sweden) connected with ÄKTA avant (GE Healthcare, United Kingdom). Refolded L-asparaginase was loaded into the pre-equilibrated column [50 mM Tris–HCl buffer, 10% (v/v) glycerol at pH 8.5] at flow rate of 1 ml/min, and the column was washed with three column volumes of refolding buffer [50 mM Tris–HCl buffer, 10% (v/v) glycerol at pH 8.5]. Recombinant L-asparaginase was eluted from the column using 0 to 0.5 M continuous gradient of NaCl. Eluates (5 ml each) containing protein were pooled and concentrated to 5 ml using a concentrator (Pall^®^ Centrifugal Devices, Pall Corporation, Puerto Rico) with the membrane of 10 kDa molecular weight cutoff. DEAE-purified concentrated eluates and refolded L-asparaginase were centrifuged (12,000 rpm, 4°C, 10 min) and analyzed on SDS-PAGE.

The size exclusion column Superdex 200 PG (GE Healthcare, United Kingdom) connected with ÄKTA avant (GE Healthcare, United Kingdom) was washed with a column volume of MQ water and equilibrated with two column volumes of 20 mM Tris–HCl, 10% (v/v) glycerol at pH 8.5. The concentrated sample (5 ml) was loaded onto the equilibrated column and eluted in 120 ml of 50 mM Tris–HCl. Washing, equilibration, and elution were performed at a flow rate of 0.5 ml/min. The quality of the purified protein was checked on SDS-PAGE. The eluted protein was concentrated to 3 ml using a concentrator (Pall^®^ Centrifugal Devices, Pall Corporation, Puerto Rico) with the membrane of 10 kDa molecular weight cutoff.

### Characterization of Purified hGH and L-Asparaginase

#### Determination of the Secondary and Tertiary Structures of Purified hGH and L-Asparaginase

The secondary structure of purified hGH and L-asparaginase was characterized using far-UV circular dichroism (CD) spectroscopy (Jasco-700 spectropolarimeter). The solutions were taken in a 1 mm path length cuvette at 20°C. Spectra were recorded from 195 to 250 nm for 200 μg/ml solution of purified hGH and L-asparaginase with that of commercial hGH and L-asparaginase in 20 mM Tris–HCl, pH 8.5, respectively. The CD spectrum was scanned three times and the average spectrum was plotted eventually.

The tertiary structure of purified hGH was characterized using tryptophan fluorescence in a Cary Eclipse spectrofluorometer (Varian, United States). Similarly, the tertiary structure of purified L-asparaginase was characterized using tryptophan fluorescence in a FluoroMax-4 spectrofluorometer (HORIBA Scientific, France). Purified hGH and commercial hGH of 200 μg/ml in 20 mM Tris–HCl were excited at 280 nm separately, and the emission spectra were recorded from 300 to 400 nm using excitation and emission slit width set at 5 nm. Similarly, the experiment was performed with purified L-asparaginase and commercial L-asparaginase of 200 μg/ml in 20 mM Tris–HCl. The fluorescence spectrum was scanned for three times and the average spectrum was plotted for analysis.

#### Bioactivity Assay of Purified hGH

Bioactivity of hGH was estimated through Nb-2 rat lymphoma cell line-based proliferation assay. Nb-2 cells were maintained in RPMI media with 5% FBS and 5% HS till the appropriate cell number is achieved; 0.1 × 10^6^ Nb-2 cells were added to each well of a flat bottom 96-well plate and incubated for 14 h in RPMI media without serum to arrest the cells in G_0_/G_1_ phase. Bioactivity of purified hGH was tested using commercial hGH as a positive control and unrelated protein bovine serum albumin (BSA) as a negative control. Two hundred nanograms per milliliter of purified hGH in RPMI media with 5% FBS and 5% HS was added to the test wells. Two hundred nanograms per milliliter of commercial hGH and 200 ng/ml of (BSA) were added to the control wells. Another group of untreated cells were maintained to compare with basal Nb-2 cell growth. Cells were incubated for 5 days at 37°C in a CO_2_ incubator, and cell numbers were calculated from each well using a hemocytometer. Statistical comparisons among groups (*n* = 3) were calculated using two-way ANOVA test (*p* < 0.05) in GraphPad Prism software.

#### Enzyme Activity of Purified L-Asparaginase

Enzymatic activity of L-asparaginase was estimated by quantification of ammonia released upon conversion of L-asparagine into aspartic acid using Nessler’s reagent (El-Naggar et al., 2018). The enzyme assay mixture consisted of 900 μl of freshly prepared L-asparagine (25 mM) in 50 mM Tris–HCl buffer (pH 8.0), 10% glycerol, and 100 μl of purified L-asparaginase after the freeze–thaw method (8 mg/ml). The reaction mixture was then incubated at 37°C for 10 min. The reaction was stopped by adding 100 μl of 1.5 M trichloroacetic acid. The precipitated protein was centrifuged at 12,000 rpm for 3 min and the ammonia released was collected in the supernatant. Then, 100 μl of supernatant was diluted in 800 μl of distilled water and used for colorimetric determination by nesslerizing with 100 μl Nessler’s reagent. The yellow-orange color thus produced was measured at 450 nm. The amount of ammonia liberated in the reaction was determined based on the standard curve obtained with ammonium chloride. One unit of L-asparaginase activity is defined as the amount of the enzyme that liberates 1 μmol of ammonia per min at 37°C and pH 8.5.

### Quality Attributes of the Freeze–Thaw Method Using hGH

Different biophysical and biochemical tools were used to investigate the molecular and structural level information of hGH IB solubilization using the freeze–thaw method.

#### Far-UV CD Spectroscopy of hGH in Solubilized State

The far-UV CD spectra of hGH were recorded using a spectropolarimeter (Jasco-700, United States) in the wavelength range of 200–250 nm at 20°C; 200 μg/ml solution of purified hGH incubated in 8-M urea-based and freeze–thaw-based buffers in 1:10 ratio was centrifuged at 12,000 rpm, 4°C for 30 min, and the supernatants were taken in a 1-mm path length cuvette. The average of three independent spectra for each sample was acquired and used for analysis.

#### Fluorescence Spectroscopy of hGH in Solubilized State

Fluorescence emission spectra were recorded using the Cary Eclipse spectrofluorometer (Varian, United States); 200 μg/ml solution of purified hGH incubated in 8-M urea-based and freeze–thaw-based buffers at 1:10 ratio was centrifuged at 12,000 rpm, 4°C for 30 min and the supernatants were excited at 280 nm. The emission spectra of solubilized proteins were then collected from 300 to 400 nm with excitation and emission slit width set at 5 nm. The average of three independent spectra for each sample was acquired and used for analysis.

#### High-Performance Liquid Chromatography of hGH in Refolded State

hGH IBs were solubilized in three different ways: (a) 10 μl of hGH IBs were solubilized in 89 μl of 2 M urea and 1 μl of 100 mM DTT, (b) 10 μl of hGH IBs were solubilized in 90 μl of 2 M urea, and (c) 10 μl of hGH IBs were solubilized in 90 μl of 8 M urea. These three suspensions were frozen overnight at −20°C and thawed at room temperature the next morning. Suspensions were centrifuged at 12,000 rpm, 4°C for 30 min. Each of the supernatants was diluted in a pulsatile manner in 900 μl of refolding buffer at 4°C containing 20 mM Tris–HCl. Refolded protein was centrifuged at 12,000 rpm, 4°C for 30 min. All these three protein samples along with commercial hGH were concentrated to 6 mg/ml using concentrators (Amicon^®^ Ultra 0.5 ml Centrifugal Filters, Merck, United States). Concentrated samples were centrifuged again at 12,000 rpm, 4°C for 10 min. Twenty microliters of each sample was loaded onto pre-equilibrated (20 mM Tris–HCl and 100 mM NaCl) HPLC column TSK gel G3000SWxl (Tosoh Biosciences, Japan) using the Waters HPLC system. The graphs of different protein samples were overlaid and the area under the curve was determined.

#### Native PAGE Analysis of hGH in Different Refolding Concentrations

One-milliliter hGH IBs were prepared from 1 L of bacterial culture and solubilized using the freeze–thaw method (2 M urea + 1 mM DTT and frozen overnight followed by thawing at room temperature the next morning). Solubilized protein after centrifugation (12,000 rpm, 30 min, 4°C) was subjected to pulsatile refolding (0.5 ml/min) with four different dilutions—initial to final volume: (a) 1:2 (1.97 mg/ml), (b) 1:5 (800 μg/ml), (c) 1:10 (380 μg/ml), and (d) 1:20 (230 μg/ml). All four dilutions were again centrifuged (12,000 rpm, 30 min, 4°C) and the supernatants were collected for native PAGE gel. Native PAGE gel was run for all these concentrations in equivalent dilution (sample d was loaded neat, sample c was loaded half diluted, sample b was loaded one-fourth diluted, and sample a was loaded 1/10th diluted in native dye) along with sample d prepared in 5 × denaturing dye for comparison of monomeric species. Sample c was selected for further time point-based study.

#### Size Exclusion Chromatography of hGH in Refolded State

Refolded hGH protein after solubilization in freeze–thaw-based buffer in the presence of DTT was incubated at 4°C for different time intervals, i.e., 1, 4, 8, 16, and 24 h. The refolded protein after every time interval was centrifuged at 12,000 rpm, 4°C for 30 min, and 4 ml of the supernatant was loaded onto 20 mM Tris–HCl pre-equilibrated size exclusion chromatographic column Superdex 200 PG 16/600 (GE Healthcare, United Kingdom) connected with ÄKTA prime (GE Healthcare, United Kingdom). Washing, equilibration, and elution were performed at a flow rate of 2 ml/min. The graphs from different time intervals were overlaid and the area under the curve was determined.

### Quality Attributes of the Freeze–Thaw Method Using L-Asparaginase

Different biophysical and biochemical tools were used to investigate the molecular and structural level information of L-asparaginase IB solubilization using the freeze–thaw method.

#### Far-UV CD Spectroscopy of L-Asparaginase in Solubilized State

The far-UV CD spectra of L-asparaginase were recorded using the spectropolarimeter (Jasco-700, United States) in the wavelength range of 200–250 nm at 20°C; 200 μg/ml solution of purified L-asparaginase incubated in 8-M urea-based and freeze–thaw-based buffers in 1:10 ratio was centrifuged at 12,000 rpm, 4°C for 30 min, and the supernatants were taken in a 1-mm path length cuvette. The average of three independent spectra for each sample was acquired and used for analysis.

#### Fluorescence Spectroscopy of L-Asparaginase in Solubilized State

Fluorescence emission spectra were recorded using the FluoroMax-4 spectrofluorometer (HORIBA Scientific, France); 200 μg/ml solution of purified L-asparaginase incubated in 8-M urea-based and freeze–thaw-based buffers at 1:10 ratio was centrifuged at 12,000 rpm, 4°C for 30 min, and the supernatants were excited at 280 nm. The emission spectra of solubilized proteins were then collected from 300 to 400 nm with excitation and emission slit width set at 5 nm. The average of three independent spectra for each sample was acquired and used for analysis.

#### High-Performance Liquid Chromatography of L-Asparaginase in Refolded State

L-Asparaginase IBs were solubilized in three different ways: (a) 10 μl of L-asparaginase IBs were solubilized in 89 μl of 2 M urea and 1 μl of 100 mM DTT, (b) 10 μl of L-asparaginase IBs were solubilized in 90 μl of 2 M urea, and (c) 10 μl of L-asparaginase IBs were solubilized in 90 μl of 8 M urea. These three suspensions were frozen overnight at −20°C and thawed at room temperature the next morning. Suspensions were centrifuged at 12,000 rpm, 4°C for 30 min. Each of the supernatants was diluted in a pulsatile manner in 900 μl of refolding buffer at 4°C containing 20 mM Tris–HCl. The refolded protein was centrifuged at 12,000 rpm, 4°C for 30 min. All these three protein samples along with commercial L-asparaginase were concentrated to 2 mg/ml using concentrators (Amicon^®^ Ultra 0.5 ml Centrifugal Filters, Merck, United States). The concentrated samples were centrifuged again at 12,000 rpm, 4°C for 10 min; 20 μl of each sample was loaded onto pre-equilibrated (20 mM Tris–HCl and 100 mM NaCl) HPLC column TSK gel G3000SWxl (Tosoh Biosciences, Japan) using the Waters HPLC system. The graphs of different protein samples were overlaid and the area under the curve was determined.

#### Optimal Concentration and Stability of L-Asparaginase Enzyme

The optimum enzyme concentration for the activity assay was determined by incubating different concentrations of purified enzyme (2–400 μg/ml) in 25 mM L-asparagine substrate concentration. The reaction mixture was then incubated at 37°C for 10 min, and later, the reaction was stopped with 1.5 M TCA. The amount of ammonia liberated upon catalytic reaction was quantified using Nessler’s reagent, and thus, optimal enzyme concentration was determined.

The stability of L-asparaginase was determined by preincubating 50 μg/ml enzyme (without its substrate) in PBS at pH 7.4 in shaking condition (200 rpm, 37°C) for different time intervals, i.e., 1, 3, 6, 12, 24, 48, and 96 h. After the end of the incubation periods, the enzyme was cooled, and the residual activities were assayed using the previously mentioned method.

### Desalting and Buffer Exchange of Refolded L-Asparaginase

HiTrap^®^ Desalting 5 ml column (GE Healthcare, United Kingdom) connected with ÄKTA avant (GE Healthcare, United Kingdom) was used for the removal of 10% (v/v) glycerol and to exchange the buffer of refolded and purified L-asparaginase with 20 mM Tris–HCl. For that, the column was washed with five column volumes of water. The column was equilibrated with two column volumes of 20 mM Tris–HCl; 1.5 ml of refolded L-asparaginase in 50 mM Tris–HCl and 10% (v/v) glycerol at pH 8.5 was loaded onto the column and subsequently eluted. The eluted fractions were collected and pooled. Washing, equilibration, and elution were performed at a flow rate of 4 ml/min.

### SDS-PAGE Analysis of Proteins

A vertical mini-gel SDS-PAGE apparatus (Bio-Rad Laboratories, United States) was used for the separation of proteins based on molecular weight. The glass plates contained 5% stacking gel and the resolving gel concentration was 12%. Based on the concentration of protein, the samples were prepared in either 2 × loading dye [125 mM Tris–HCl at pH 6.8, 20% (v/v) glycerol, 4% (w/v) SDS, 10% (v/v) β-mercaptoethanol, and 0.2% (w/v) bromophenol blue] or 5 × loading dye [125 mM Tris–HCl at pH 6.8, 50% (v/v) glycerol, 10% (w/v) SDS, 25% (v/v) β-mercaptoethanol, and 0.2% (w/v) bromophenol blue]. The samples in non-reducing condition were prepared with 5 × non-reducing dye [125 mM Tris–HCl at pH 6.8, 50% (v/v) glycerol, 10% (w/v) SDS, and 0.2% (w/v) bromophenol blue]. All the protein samples loaded onto the gel were prepared in equivalent dilution. The protein bands were visualized by staining in a staining solution [0.2% (w/v) Coomassie Brilliant Blue R-250, 10% (v/v) glacial acetic acid, and 50% (v/v) methanol] followed by destaining with a destaining solution [10% (v/v) glacial acetic acid and 50% (v/v) methanol].

### Native PAGE Analysis of Proteins

The vertical mini-gel apparatus (Bio-Rad Laboratories, United States) was used for the separation of proteins based on net charge, size, and shape at 4°C. The glass plates were wiped with ethanol to avoid any residual denaturing agent and contained 5% stacking gel and 12% resolving gel. The samples were prepared in 4 × native dye [125 mM Tris–HCl at pH 6.8, 40% (v/v) glycerol and 0.2% (w/v) bromophenol blue]. All the protein samples loaded onto the gel were prepared in equivalent dilution. The protein bands were visualized by staining in a staining solution [0.2% (w/v) Coomassie Brilliant Blue R-250, 10% (v/v) glacial acetic acid, and 50% (v/v) methanol] followed by destaining with a destaining solution [10% (v/v) glacial acetic acid and 50% (v/v) methanol].

### Protein Estimation by BCA

Protein samples obtained from different processing steps, viz. IBs, solubilized, refolded, and purified proteins, were estimated using the BCA assay kit. BSA was taken as a standard protein and its varying concentrations (2,000–31.25 μg/ml) were prepared with serial dilution in a 96-well plate. Samples of IBs and solubilized protein were diluted 100 and 10 times, respectively, while the rest of the samples were undiluted. The concentration of each sample was estimated by measuring OD at 575 nm. Finally, the amount of each protein sample was calculated, and the step yield and overall yield were analyzed.

## Results

### Refolding of Recombinant hGH and L-Asparaginase Expressed as Inclusion Bodies

**FIGURE 1:**
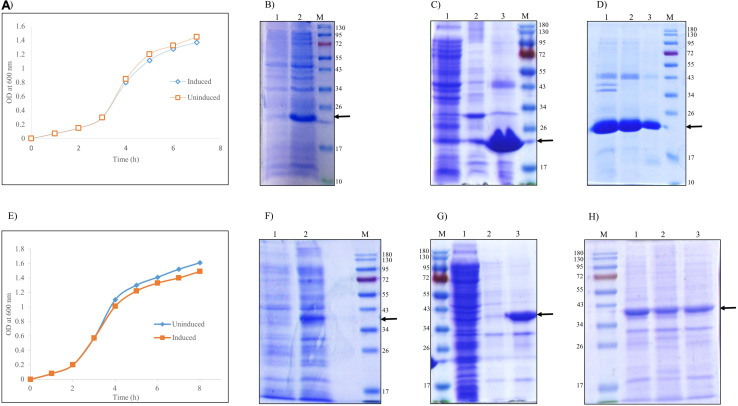
Refolding of recombinant human growth hormone and L-asparaginase expressed as inclusion bodies (IBs) in *Escherichia coli.* Arrow indicates the protein of interest. Lane M represents molecular weight marker (180, 130, 95, 72, 55, 43, 34, 26, 17, and 10 kDa). **(A)** Growth kinetics of uninduced and induced culture of *E. coli* expressing r-hGH. **(B)** SDS-PAGE analysis of human growth hormone (hGH) expression: lane 1, uninduced cell lysate; lane 2, induced cell lysate; lane M, molecular weight marker. **(C)** SDS-PAGE analysis of isolated inclusion bodies of hGH: lane 1, supernatant of cell lysate; lane 2, water wash of IBs; lane 3, isolated hGH IBs (MW 22 kDa); lane M, molecular weight marker. **(D)** SDS-PAGE analysis of solubilization and refolding of recombinant hGH from inclusion body aggregates: lane 1, hGH IBs; lane 2, hGH IBs solubilized in freeze–thaw-based buffer; lane 2, refolded hGH; lane M, molecular weight marker. **(E)** Growth kinetics of uninduced and induced culture of *E. coli* expressing recombinant L-asparaginase. **(F)** SDS-PAGE analysis of recombinant L-asparaginase expression: lane 1, uninduced cell lysate; lane 2, induced cell lysate; lane M, molecular weight marker. **(G)** SDS-PAGE analysis of isolated inclusion bodies of L-asparaginase: lane M, molecular weight marker; lane 1, supernatant of cell lysate; lane 2, water wash of IBs; lane 3, isolated asparaginase IBs (MW 37 kDa). **(H)** SDS-PAGE analysis of solubilization and refolding of recombinant L-asparaginase from inclusion body aggregates: lane M, molecular weight marker; lane 1, L-asparaginase IBs; lane 2, L-asparaginase IBs solubilized in freeze–thaw-based buffer; lane 3, refolded L-asparaginase.

*E. coli* cells grown in LB medium were induced with IPTG for expression of r-hGH and L-asparaginase. Lowering of cell growth after induction with IPTG in comparison with the uninduced culture indicated the production of recombinant protein (Figures 1A,E). Cell pellets of uninduced and induced culture were analyzed by SDS-PAGE for the expression of recombinant hGH (Figure 1B) and L-asparaginase (Figure 1F). hGH and L-asparaginase were expressed intracellularly as aggregates having molecular weights of 22 and 37 kDa, respectively.

Bacterial cell pellet resuspended in lysis buffer was homogenized, sonicated, and centrifuged. Most of the expressed hGH (Figure 1C) and L-asparaginase (Figure 1G) proteins were in pellet fraction as IBs, whereas very little hGH and L-asparaginase were in the supernatant. No significant loss of hGH and L-asparaginase IBs occurred during the washing step. Recombinant hGH and L-asparaginase expressed as IBs were almost 90% pure as evident from the SDS-PAGE analysis. Purified IB aggregates were used for solubilization and refolding studies. Solubilization and refolding experiments were always carried out with freshly prepared IBs.

Since inclusion body aggregates lack aqueous solubility, isolated hGH and L-asparaginase IBs were solubilized using the freeze–thaw-based method reported earlier (Qi et al., 2015). Efficient refolding majorly depends upon reducing the intermolecular interactions of protein intermediates, thereby minimizing the possibility of protein aggregation. Solubilized protein was thus refolded by pulsatile dilution in the respective refolding buffers [50 mM Tris–HCl, pH 8.5 for solubilized hGH and 50 mM Tris–HCl, 10% (v/v) glycerol, pH 8.5 for solubilized L-asparaginase] at 4°C with constant stirring. Inclusion bodies (IBs), the solubilized protein, and the refolded protein were analyzed by SDS-PAGE (Figures 1D,H).

### Purification of Refolded Proteins

Although refolded hGH and L-asparaginase after the freeze–thaw-based solubilization were almost 90% pure, ion exchange and size exclusion chromatography were used to further purify the proteins.

#### Purification of Refolded hGH

**FIGURE 2:**
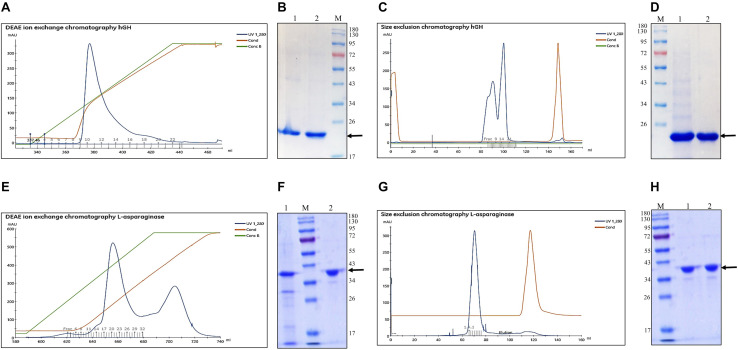
Purification of refolded human growth hormone and L-asparaginase after solubilization in freeze–thaw-based method. Arrow indicates the protein of interest. Lane M represents molecular weight marker (180, 130, 95, 72, 55, 43, 34, 26, 17, and 10 kDa). **(A)** Diethylaminoethyl (DEAE) anion exchange chromatogram for purification of refolded hGH after solubilization in freeze–thaw buffer. **(B)** SDS-PAGE analysis of DEAE ion exchange eluted fractions: lane 1, refolded hGH; lane 2, pooled fractions of DEAE-purified hGH; lane M, molecular weight marker. **(C)** Size exclusion chromatogram (SEC) for purification of hGH after DEAE ion exchange chromatography. **(D)** SDS-PAGE analysis of SEC fractions: lane M, molecular weight marker; lane 1, pooled and concentrated DEAE-purified hGH; lane 2, SEC-purified hGH. **(E)** DEAE anion exchange chromatogram for purification of refolded L-asparaginase after solubilization in freeze–thaw buffer. **(F)** SDS-PAGE analysis of DEAE ion exchange eluted fractions: lane 1, refolded L-asparaginase; lane M, molecular weight marker; lane 2, pooled and concentrated DEAE-purified L-asparaginase. **(G)** SEC for purification of L-asparaginase after DEAE ion exchange chromatography. **(H)** SDS-PAGE analysis of SEC fractions: lane M, molecular weight marker; lane 1, pooled and concentrated DEAE-purified L-asparaginase; lane 2, SEC-purified L-asparaginase.

DEAE ion exchange chromatography was selected for the purification of hGH. The refolded protein was purified by gradient elution using NaCl and the chromatogram is presented in Figure 2A. The blue, green, and maroon lines in the elution profile depict the protein, gradient of NaCl, and conductance, respectively. The fractions under the peak area were pooled and loaded onto SDS-PAGE and analyzed (Figure 2B). The eluted fractions of hGH from DEAE ion exchange chromatography were further concentrated. The concentrated protein sample was centrifuged to remove protein aggregates and loaded onto Superdex 75 PG 16/600 size exclusion chromatographic column (resolution range: 3–70 kDa). The chromatograms depicted in Figure 2C show the elution profile with a minor and a major peak. The major peak represents monomeric hGH, whereas the minor peak represents dimeric hGH (Nagatomi et al., 2000). Since dimeric hGH is known to have lower biological activity compared with monomeric hGH (Becker et al., 1987), the fractions of monomeric hGH were collected and finally analyzed on SDS-PAGE which was found to be highly pure (Figure 2D).

The step yield and overall yield of each processing step to recover the purified protein were estimated using the BCA assay (Table 1). Each processing step had considerable efficiency except for the refolding step that costed more than 75% of the total hGH protein. Overall yield of the purified protein was found to be 14% using freeze–thaw as a solubilization method for hGH IBs.

**TABLE 1:** Purification of recombinant hGH from bacterial inclusion bodies using freeze–thaw as a solubilization method.

Steps	Total protein (mg)	Step yield (%)	Overall yield (%)
Inclusion bodies	72.19	100	100
Solubilization	71.97	99.7	99.7
Refolding	24.11	33.5	33.4
DEAE ion exchange chromatography	16.07	66.6	22.3
Size exclusion chromatography	9.76	60.7	13.5

**DEAE, diethylaminoethyl; hGH, human growth hormone.**

#### Purification of Refolded L-Asparaginase

Refolded L-asparaginase protein was purified using DEAE ion exchange chromatography and the chromatogram is presented in Figure 2E. The fractions under the major peak area were pooled and loaded onto SDS-PAGE (Figure 2F). It was observed that protein contamination of lower molecular weight than L-asparaginase got eliminated after DEAE ion exchange chromatography. The fractions under the minor peak were omitted due to increasing protein contamination with respect to L-asparaginase. The eluted fractions of L-asparaginase under the major peak from the chromatogram were further concentrated and centrifuged. The concentrated protein sample was finally loaded onto Superdex 200 PG 16/600 size exclusion chromatographic column (resolution range: 10–600 kDa). The chromatograms depicted in Figure 2G shows the elution profile with a single peak which represents tetrameric L-asparaginase with a molecular weight around 150 kDa. The fractions of size exclusion chromatography purified L-asparaginase were pooled and finally found to be highly pure on SDS-PAGE (Figure 2H).

The step yield and overall yield of each processing step to recover the purified protein were estimated using the BCA assay (Table 2). Each processing step had considerable efficiency except the refolding and DEAE ion exchange chromatography step that costed around 70% of the total L-asparaginase protein. Overall yield of the purified protein was found to be around 25% using freeze–thaw as a solubilization method for L-asparaginase IBs.

**TABLE 2:** Purification of recombinant L-asparaginase from bacterial inclusion bodies using freeze–thaw as a solubilization method.

Steps	Total protein (mg)	Step yield (%)	Overall yield (%)
Inclusion bodies	88	100	100
Solubilization	85	96.6	96.6
Refolding	44	51.8	50
DEAE ion exchange chromatography	23.5	53.4	26.7
Size exclusion chromatography	22.35	95.1	25.4

### Characterization of Purified hGH

Refolded and purified hGH proteins after solubilization in the freeze–thaw-based buffer were used for detailed biophysical and biological characterization.

#### Secondary and Tertiary Structures of hGH

**FIGURE 3:**
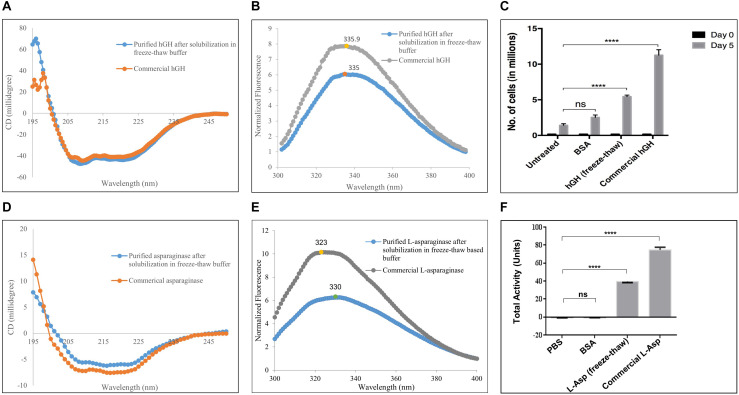
Characterization of purified hGH and L-asparaginase after solubilization in freeze–thaw-based method. **(A)** Secondary structure determination of purified hGH by far-UV circular dichroism spectroscopy in comparison with commercial hGH. **(B)** Tertiary structure determination of purified hGH by fluorescence spectroscopy in comparison with commercial hGH. **(C)** Nb-2 cell line-based bioactivity assay of purified hGH: untreated, no hGH added in the medium; BSA, bovine serum albumin added in the medium as a negative control; hGH, purified hGH after solubilization in freeze–thaw buffer; commercial hGH, commercially purchased bioactive hGH for positive control; day 0, the initial number of cells in millions; day 5, the final number of cells in millions. The concentration of BSA, commercial hGH, and purified hGH was 200 ng/ml. **(D)** Secondary structure determination of purified L-asparaginase by far-UV circular dichroism spectroscopy in comparison with commercial asparaginase. **(E)** Tertiary structure determination of purified L-asparaginase by fluorescence spectroscopy in comparison with commercial L-asparaginase. **(F)** Nessler’s reagent-based enzyme activity of purified L-asparaginase: PBS, phosphate buffer saline; BSA, bovine serum albumin added in the medium as a negative control; L-asparaginase (freeze–thaw), purified L-asparaginase after solubilization in freeze–thaw buffer; commercial L-asparaginase, commercially purchased active L-asparaginase for positive control. “****” on the bar graphs indicates that there was significant difference of *p* value less than 0.05 when compared with the control.

The presence of a secondary structure in purified hGH was confirmed by far-UV CD spectroscopy (Figure 3A). Protein samples were kept in 20 mM Tris–HCl to avoid any hike in voltage in the photomultiplier tube (PMT). hGH possesses 10 α-helices in its native structure (UniProt ID: P01241). The presence of α-helices is characterized by the negative bands at 208 and 222 nm in far-UV CD spectra as demonstrated in Figure 3A. The purified hGH from the freeze–thaw-based buffer aligned with the commercial sample of hGH which confirms the integrity of the secondary structure in purified hGH.

The tertiary structure of purified hGH as determined by fluorescence spectroscopy is shown in Figure 3B. hGH contains a single tryptophan residue and eight tyrosine residues as intrinsic fluorophores (UniProt ID: P01241). In an unfolded state, tryptophan and tyrosine residues show maxima at 357 and 305 nm, respectively. However, in a folded state, tryptophan and tyrosine residues are buried in the hydrophobic core of the protein together (Royer, 2006). This prompts fluorescence resonance energy transfer between both residues and, thus, emits at an intermediate wavelength, around 337 nm, upon excitation at 280 nm (Lakowicz, 2006; Upadhyay et al., 2016). From Figure 3B, it was observed that the purified hGH using the freeze–thaw buffer gave the maxima around 335 nm. Furthermore, the commercially purchased bioactive hGH has also aligned with the purified hGH which signifies that the purified hGH has an intact tertiary structure.

#### Bioactivity of Purified hGH

Since human growth hormone (hGH) promotes cellular proliferation by binding to prolactin receptor on the human cell, its bioactivity was confirmed by its ability to promote proliferation of mammalian cells. The Nb-2 cell line, a rat lymphoma cell line, has prolactin receptors on the cell surface that internalizes on prolactin binding and induces tumorigenesis (Ali et al., 1991). hGH binding to prolactin receptor on mammalian cell surface also induces mitogenesis. The increase in cell number, thus, was used as a parameter to measure the bioactivity of hGH (Bozzola et al., 1998). Figure 3C shows the bioactivity assay of purified hGH obtained after solubilization in the freeze–thaw-based buffer. Untreated cells were taken to compare cellular proliferation without any inducer. Cells treated with an unrelated protein, bovine serum albumin, served as a negative control, which ensures that cells cannot proliferate because of any protein present outside of the cells. Purified hGH from the freeze–thaw buffer showed comparable proliferation of cells at a concentration of 200 ng/ml, and the increase in cell number was significantly higher than that observed in the control groups. However, commercial hGH had higher bioactivity than purified hGH from the freeze–thaw method also.

### Characterization of Purified L-Asparaginase

Refolded L-asparaginase and purified L-asparaginase after solubilization in the freeze–thaw-based buffer were used for detailed biophysical and biochemical characterization.

#### Secondary and Tertiary Structures of L-Asparaginase

The presence of a secondary structure in purified L-asparaginase was confirmed by far-UV CD spectroscopy (Figure 3D). L-Asparaginase possesses 15 α-helices in each monomeric unit of its native tetrameric structure (UniProt ID: P00805) which can be observed by the negative bands at 208 and 222 nm in far-UV CD spectra as demonstrated in Figure 3D. The purified L-asparaginase from the freeze–thaw-based buffer aligned with the commercial sample of L-asparaginase which confirms the presence of a secondary structure in purified L-asparaginase.

The tertiary structure of purified L-asparaginase as determined by fluorescence spectroscopy is shown in Figure 3E. Each monomeric unit of L-asparaginase contains a single tryptophan residue and 12 tyrosine residues as intrinsic fluorophores (UniProt ID: P00805). In a folded state, the fluorescence emission spectrum of L-asparaginase shows maxima at about 320 nm, while the emission intensity is reduced to one-third with a red shift to about 350 nm in case of a completely unfolded state of L-asparaginase (Upadhyay et al., 2014; Verma et al., 2014). From Figure 3E, the commercial L-asparaginase showed emission maximum at 323 nm. However, purified L-asparaginase using the freeze–thaw method showed a decrease in emission maxima to about half of that of commercial L-asparaginase. Also, a slight red shift in emission maxima to 330 nm was observed as compared with commercial L-asparaginase. Both observations suggest that purified L-asparaginase is partially unfolded.

#### Enzyme Activity of Purified L-Asparaginase

Since tetrameric L-asparaginase catalyzes L-asparagine into aspartate and ammonia, its enzymatic activity was confirmed by its estimation of ammonia released using Nessler’s reagent. Figure 3F shows the enzyme activity assay of purified L-asparaginase obtained after solubilization in the freeze–thaw-based buffer. PBS was taken as a negative control to compare the total activity without any protein. A reaction mixture with an unrelated protein, bovine serum albumin, served as a negative control in order to ensure that any protein present in the mixture cannot possess total activity. Purified and commercial L-asparaginase from the freeze–thaw buffer showed comparable total activity, and it was significantly higher than that observed with the control groups. However, commercial L-asparaginase had higher enzymatic activity than purified L-asparaginase from the freeze–thaw method.

### Quality Attributes of the Freeze–Thaw Method Using hGH

**FIGURE 4:**
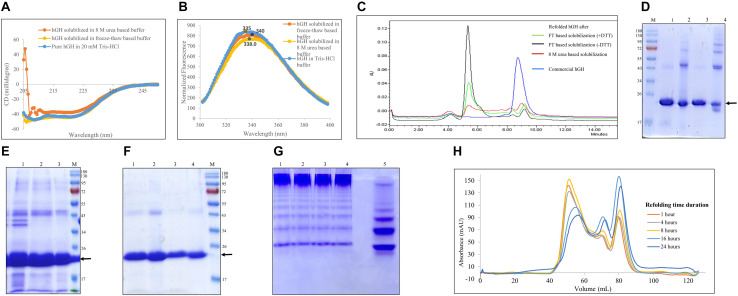
Quality attributes of the freeze–thaw method using hGH protein. Arrow indicates the protein of interest. Lane M represents molecular weight marker (180, 130, 95, 72, 55, 43, 34, 26, 17, and 10 kDa). **(A)** Analysis of secondary structure of hGH in different solubilized states: far-UV CD spectra of hGH in freeze–thaw-based and 8-M urea-based solubilization buffers and that of pure hGH in 20 mM Tris–HCl buffer. **(B)** Analysis of the tertiary structure of hGH in different solubilization buffers: fluorescence spectra of hGH incubated in freeze–thaw-based and 8-M urea-based solubilization buffers and in 20 mM Tris–HCl buffer. **(C)** Analysis of concentrated refolded hGH species (6 mg/ml) by high-performance liquid chromatography (HPLC): refolded hGH after FT-based solubilization (+ DTT), refolded hGH after freeze–thaw-based solubilization buffer in the presence of dithiothreitol (DTT); refolded hGH after FT-based solubilization (-DTT), refolded hGH after freeze–thaw-based solubilization buffer in the absence of DTT; refolded hGH after 8-M urea-based buffer; commercial hGH. **(D)** Analysis of concentrated refolded hGH species (6 mg/ml) by SDS-PAGE: lane M, molecular weight marker; lane 1, refolded hGH in a reducing dye after freeze–thaw-based solubilization buffer in the presence of DTT; lane 2, refolded hGH in a non-reducing dye after freeze–thaw-based solubilization buffer in the presence of DTT; lane 3, refolded hGH in a reducing dye after freeze–thaw-based solubilization buffer in the absence of DTT; lane 4, refolded hGH in a non-reducing dye after freeze–thaw-based solubilization buffer in the absence of DTT. **(E)** SDS-PAGE analysis of hGH solubilization: lane 1, hGH IBs; lane 2, solubilized hGH in the presence of DTT; lane 3, solubilized hGH in the absence of DTT. **(F)** Analysis of refolded hGH species by SDS-PAGE analysis: lane 1, refolded hGH (400 μg/ml) in a reducing dye after freeze–thaw-based solubilization buffer in the presence of DTT; lane 2, refolded hGH (400 μg/ml) in a non-reducing dye after freeze–thaw-based solubilization buffer in the presence of DTT; lane 3, refolded hGH (250 μg/ml) in a reducing dye after freeze–thaw-based solubilization buffer in the absence of DTT; lane 4, refolded hGH (250 μg/ml) in a non-reducing dye after freeze–thaw-based solubilization buffer in the absence of DTT; lane M, molecular weight marker. **(G)** Native PAGE analysis of different concentrations of refolded proteins: lane 1, refolded protein after 1:2 dilution (2,000 μg/ml); lane 2, refolded protein after 1:5 dilution (800 μg/ml); lane 3, refolded protein after 1:10 dilution (400 μg/ml); lane 4, refolded protein after 1:20 dilution (200 μg/ml); lane 5, refolded protein after 1:20 dilution (200 μg/ml). Samples of lanes 1–4 are prepared in native dye (absence of SDS and β-mercaptoethanol), while the lane 5 sample is prepared in denaturing dye (presence of SDS and β-mercaptoethanol). **(H)** Size exclusion chromatography analysis of hGH in refolded state using different time intervals, i.e., 1, 4, 8, 16, and 24 h.

The solubilization buffer based on the freeze–thaw method demonstrated very high solubilization efficiency of IB aggregates but low protein recovery during refolding. To get insights into how the freeze–thaw-based buffer solubilizes protein, the secondary and tertiary structures of hGH in the buffer were analyzed using circular dichroism and fluorescence spectroscopy, respectively (Figures 4A,B). Figures 4C,E demonstrate the effect of reducing agent like DTT in freeze–thaw buffer on refolding and solubilization efficiency of hGH using HPLC and SDS-PAGE, respectively (Table 3). Figures 4D,F present the effect of concentration after refolding of protein in the presence and absence of DTT using SDS-PAGE. Figures 4G,H demonstrate the effect of refolding concentration and kinetics using native PAGE and size exclusion chromatography, respectively (Tables 4, 5).

**TABLE 3:** Percentage population of refolded hGH species after subjecting to different types of solubilization with respect to commercial hGH.

Protein samples	Population of aggregates (%)	Population of dimers (%)	Population of monomers (%)
Refolded hGH after freeze–thaw solubilization with DTT	76	4.5	17.3
Refolded hGH after freeze–thaw solubilization without DTT	89	2	7.4
Refolded hGH after solubilization in 8 M urea	39	11.7	32.5
Commercial hGH	4.6	1.4	93.4

**DTT, dithiothreitol.**

**TABLE 4:** Step yield and concentration for solubilized and varying dilution of refolded hGH proteins.

Protein samples	Step yield (%)	Concentration (mg/ml)
Solubilized protein	100	12.96
Refolded protein (1:2)	30.6	1.97
Refolded protein (1:5)	30.8	0.8
Refolded protein (1:10)	29.3	0.38
Refolded protein (1:20)	35.46	0.23

**TABLE 5:** Percentage population of different hGH species after incubating refolded hGH for different time durations.

Refolding time duration (h)	Population of aggregates (%)	Population of dimers (%)	Population of monomers (%)
1	59	15.3	25.7
4	61.5	13.4	25
8	61.6	13.9	24.5
16	39.1	19.6	41.3
24	44.5	17.5	37.9

#### Biophysical Analysis of hGH Protein in Solubilized State

hGH has 10 α-helices (UniProt ID: P01241) in its structure. Therefore, its far-UV CD spectrum is characterized by negative peaks at 208 and 222 nm. Figure 4A shows the secondary structure of hGH in freeze–thaw-based and 8-M urea-based solubilization buffers. It was observed that hGH solubilized in different buffers was aligned to the pure hGH in Tris–HCl buffer. High salt concentration in 8-M urea-solubilized hGH caused the increment of high tension (HT) voltage with decreasing wavelength, thereby resulting in low signal to noise ratio (Greenfield, 2007).

As mentioned earlier, hGH has one tryptophan and eight tyrosine residues (UniProt ID: P01241). Having a single tryptophan residue in protein is always desirable for fluorescence studies because a multiple tryptophan system will contribute to the emission spectra differently based on their local environment and conformation, making the emission spectra very challenging to interpret (Lakowicz, 2006). Figure 4B shows the fluorescence spectra of hGH in different solubilization buffers. The spectra of hGH solubilized in urea- and freeze–thaw-based buffers looked similar to those of native protein in normal buffer with a slight red shift which confirms the integrity of the tertiary structure in the solubilized state of hGH.

#### Biochemical Analysis of Refolded hGH Species

From Table 1, it was observed that the overall protein recovery using the freeze–thaw method for hGH was significantly low. The bioactivity of purified hGH using this method was also found to be lower than that observed with commercial hGH (Figure 3C). This suggested the possible presence of soluble aggregates and/or misfolded and/or unstable protein species (Fink, 1998; Toichi et al., 2013; Saotome et al., 2019). It was hypothesized that such refolded species could be due to the presence of reducing agent like 1 mM DTT in the freeze–thaw buffer, high protein concentration, or longer refolding time duration.

The presence of DTT in the solubilization buffer is known to break the disulfide bonds in (IBs). However, disruption of S–S bonds during solubilization could lead to scrambling of intermolecular disulfide bonds and, thus, the formation of soluble aggregates during refolding (Toichi et al., 2013). Therefore, hGH IBs were solubilized in three different ways and then the refolded proteins were analyzed on HPLC column (TSK gel G3000SWxl). TSK gel G3000SWxl has 15 ml of column volume and 5 ml of void volume. Any molecule beyond its resolution range (10–500 kDa) including soluble aggregates will be first eluted in its void volume (around 5 ml) and any refolded species of hGH (22 kDa) will elute later (more than 5 ml). Figure 4C shows the elution profile of refolded hGH (6 mg/ml) solubilized in different buffers. It was observed that the peak of aggregates (eluted at 4–5 ml) was highest for freeze–thaw-based solubilization without DTT (89%), lower for freeze–thaw-based solubilization with DTT (76%) followed by 8-M urea-based solubilization (39%) and the least for commercial hGH (4.6%) (Table 3). To our surprise, this suggests that the tendency to form soluble aggregates is the highest in the freeze–thaw-based buffer without DTT. In order to check the dominating force for the formation of soluble aggregates, 15 μg of concentrated refolded hGH (6 mg/ml) after solubilization in the freeze–thaw-based buffer in the absence and presence of DTT was loaded onto SDS-PAGE in reducing and non-reducing conditions. From Figure 4D, the greater number of oligomeric bands was observed in lane 4 than in lane 2 of SDS-PAGE. This suggests that the tendency to oligomerization is higher for the solubilization buffer without DTT than that with DTT. Moreover, as demonstrated in Figure 4E, the presence of DTT in the freeze–thaw buffer resulted in higher solubilization efficiency than that without DTT in the buffer.

It is reported that the high concentration of refolded protein leads to the formation of oligomers and, thus, soluble aggregates (Fink, 1998). Thus, it was hypothesized that the formation of soluble aggregates as observed in Figures 4C,D could also be due to the high concentration of refolded protein. Therefore, the refolded protein after solubilization in the freeze–thaw-based buffer in the absence (250 μg/ml) and presence of DTT (400 μg/ml) was loaded onto SDS-PAGE in reducing and non-reducing conditions. From Figure 4F, there were no non-reducing condition-specific high molecular weight oligomers observed in lane 2 and lane 4 of SDS-PAGE. This validates that the formation of oligomers and soluble aggregates after refolding is a concentration-dependent phenomenon and the absence of DTT in the freeze–thaw buffer seems to increase the oligomerization tendency and decrease the solubilization efficiency in the case of hGH.

The formation of soluble aggregates in native condition is also reported to increase with respect to concentration and time (Proctor et al., 2010; Ciryam et al., 2015). Hence, different refolding concentrations as shown in Table 4 were analyzed for its propensity to form soluble aggregates. In contrast to the concentration-dependent aggregation phenomenon, the native gel as shown in Figure 4G revealed that varying concentrations of refolding protein had no effect on its aggregation propensity or refolding yield of monomeric protein. This was also evident to us from the BCA assay where the refolded protein yield was not getting compromised with respect to varying dilutions (Table 4). Therefore, the most commonly used dilution ratio of 1:10 was selected for further time point-based study. The possibility of time-dependent aggregation propensity of refolded hGH protein after solubilization in freeze–thaw buffer with DTT was also explored. Interestingly, Figure 4H demonstrates that the initial hours of refolding duration (1, 4, and 8 h) have the higher tendency to form soluble aggregates as compared with overnight incubation (18–24 h) of refolded hGH protein at 4°C. Alternatively, refolding yield of monomeric hGH improves by 30–40% when refolded hGH was stored overnight (Table 5).

### Quality Attributes of the Freeze–Thaw Method Using L-Asparaginase

**FIGURE 5:**
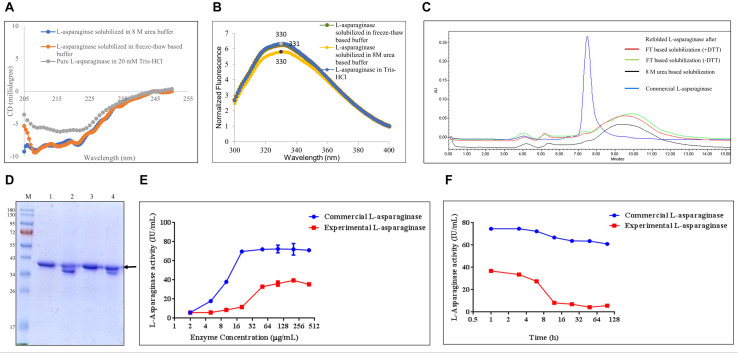
Quality attributes of the freeze–thaw method using L-asparaginase protein. Arrow indicates the protein of interest. Lane M represents molecular weight marker (180, 130, 95, 72, 55, 43, 34, 26, 17, and 10 kDa). **(A)** Analysis of the secondary structure of L-asparaginase in different solubilized states: far-UV CD spectra of L-asparaginase in freeze–thaw-based and 8-M urea-based solubilization buffers and that of pure hGH in 20 mM Tris–HCl buffer. **(B)** Analysis of the tertiary structure of L-asparaginase in different solubilization buffers: fluorescence spectra of L-asparaginase incubated in freeze–thaw-based and 8-M urea-based solubilization buffers and in 20 mM Tris–HCl buffer. **(C)** Analysis of concentrated refolded L-asparaginase species (2 mg/ml) by HPLC: refolded L-asparaginase after FT-based solubilization (+ DTT), refolded L-asparaginase after freeze–thaw-based solubilization buffer in the presence of dithiothreitol (DTT); refolded L-asparaginase after FT-based solubilization (-DTT), refolded L-asparaginase after freeze–thaw-based solubilization buffer in the absence of dithiothreitol (DTT); refolded L-asparaginase after 8-M urea-based buffer; commercial L-asparaginase. **(D)** Analysis of concentrated refolded L-asparaginase species (2 mg/ml) by SDS-PAGE: lane M, molecular weight marker; lane 1, refolded L-asparaginase in a reducing dye after freeze–thaw-based solubilization buffer in the presence of DTT; lane 2, refolded L-asparaginase in a non-reducing dye after freeze–thaw-based solubilization buffer in the presence of DTT; lane 3, refolded L-asparaginase in a reducing dye after freeze–thaw-based solubilization buffer in the absence of DTT; lane 4, refolded L-asparaginase in a non-reducing dye after freeze–thaw-based solubilization buffer in the absence of DTT. **(E)** Standardization of refolded L-asparaginase enzyme activity with respect to varying concentrations in comparison with commercial L-asparaginase. **(F)** Stability study of refolded L-asparaginase at optimum concentration with respect to time in comparison with commercial L-asparaginase.

The solubilization buffer based on the freeze–thaw method demonstrated very high solubilization efficiency of L-asparaginase IB aggregates but low protein recovery during refolding. To get insights into how the freeze–thaw-based buffer solubilizes L-asparaginase, the secondary and tertiary structures of the protein in the buffer were analyzed using circular dichroism and fluorescence spectroscopy, respectively (Figures 5A,B). Figures 5C,D demonstrate the effect of reducing agent like DTT in the freeze–thaw buffer on refolding efficiency of hGH using HPLC and SDS-PAGE, respectively (Table 6). Figure 5E presents the standardization of refolded L-asparaginase enzyme activity with respect to varying concentrations. Figure 5F demonstrates the stability study of refolded L-asparaginase at optimum concentration with respect to time.

**TABLE 6:** Percentage population of refolded L-asparaginase species after subjecting to different types of solubilization with respect to commercial L-asparaginase.

	Population of aggregates (%)	Protein species population (%)
Refolded L-asparaginase after freeze–thaw solubilization with DTT	6	93.5
Refolded L-asparaginase after freeze–thaw solubilization without DTT	7.2	92.1
Refolded L-asparaginase after solubilization in 8 M urea	5	95
Commercial L-asparaginase	17.9	81.4

#### Biophysical Analysis of L-Asparaginase Protein in Solubilized State

L-Asparaginase has 15 α-helices (UniProt ID: P00805) in each monomeric subunit of tetrameric L-asparaginase. Therefore, its far-UV CD spectrum is characterized by negative peaks at 208 and 222 nm. Figure 5A shows the secondary structure of L-asparaginase in the freeze–thaw-based and 8-M urea-based solubilization buffers. It was observed that L-asparaginase solubilized in different buffers did not align with the pure L-asparaginase in Tris–HCl buffer. Rather, the freeze–thaw-solubilized protein aligned well with the 8-M urea-solubilized protein. This demonstrates that the freeze–thaw method could not protect the secondary structure of L-asparaginase like other mild solubilization methods and behaved like a strong denaturing agent for soft inclusion body protein like L-asparaginase.

The effect of both solubilization buffers on the tertiary structure of L-asparaginase was investigated by fluorescence spectroscopy. As mentioned earlier, L-asparaginase has one tryptophan and 12 tyrosine residues in its monomeric unit (UniProt ID: P00805). Figure 5B shows the fluorescence spectra of L-asparaginase in different solubilization buffers. The spectra of L-asparaginase solubilized in 8-M urea-based and freeze–thaw-based buffers looked similar to those of purified protein in 20 mM Tris–HCl. However, as mentioned earlier, purified L-asparaginase in 20 mM Tris–HCl buffer itself was partially unfolded, and hence, the rest of the solubilized proteins were also partially unfolded.

#### Biochemical Analysis of Refolded L-Asparaginase Species

From Table 2, it was observed that the overall protein recovery using the freeze–thaw method for L-asparaginase was significantly low. The enzyme activity of purified L-asparaginase using this method was also found to be lower than that observed with commercial L-asparaginase (Figure 3F). The emission spectrum of purified L-asparaginase also indicated the presence of partially unfolded protein species (Figure 5B). This suggested the possible presence of soluble aggregates and/or misfolded and/or unstable protein species. It was thus hypothesized that such refolded species could be due to the presence of a reducing agent like 1 mM DTT in the freeze–thaw buffer, high protein concentration, or refolding time duration.

The effect of the addition of DTT in the case of L-asparaginase protein (2 mg/ml) was also investigated using HPLC. From Figure 5C, it was observed that commercial L-asparaginase was eluted at about 150 kDa which suggested the presence of native-like tetrameric L-asparaginase. The majority of the elution peak area for all the refolded protein species was observed after that of commercial L-asparaginase. This indicated the possibility of monomeric or degraded L-asparaginase or low molecular weight contaminating protein species which amounted to almost 90–95% of the population, as compared with the 81% tetrameric population of commercial L-asparaginase (Table 6). A small overlapping peak area between refolded protein species and commercial L-asparaginase indicates that a tiny fraction of tetrameric L-asparaginase was also present in refolded protein species. It was also observed from Figure 5C that all the refolded protein species after solubilization in different buffers and commercial L-asparaginase lacked soluble aggregates (Table 6). SDS-PAGE analysis in reducing and non-reducing conditions confirmed that there are no soluble aggregates present in the refolded proteins (Figure 5D).

From HPLC and SDS-PAGE analysis (Figures 5C,D), it was clear that soluble aggregates cannot be the reason for the low active protein species in the purified L-asparaginase after solubilization in the freeze–thaw buffer. The analysis also indicated the presence of monomeric refolded L-asparaginase protein species predominantly. This questioned the stability of purified L-asparaginase as tetramer with respect to time. Therefore, the enzyme concentration was optimized based on its activity as compared with commercial L-asparaginase. It was observed from Figure 5E that both purified L-asparaginase and commercial L-asparaginase reach plateau of enzyme activity after 50 μg/ml, and thus, it was further used to investigate the stability of L-asparaginase with respect to time. It was observed from Figure 5F that the stability of the enzyme decreases drastically after 6 h and enzyme activity becomes almost one-fourth of the initial time point. However, commercial L-asparaginase does not demonstrate a sharp reduction in activity even until 96 h. This indicates that the refolded L-asparaginase after the freeze–thaw solubilization method is not as stable as the commercial L-asparaginase.

## Discussion

High-throughput protein production from bacterial (IBs) has been a great challenge for bioprocess engineering. One of the main reasons for the poor recovery of functionally active protein is the suboptimal solubilization and refolding strategies (Singh et al., 2015). In the last two decades, mild solubilization methods have turned out to be a better strategy over strong denaturing buffers (Singhvi et al., 2020). The recently developed simple and economical freeze–thaw-based solubilization method has attracted many researchers in the last 5 years (Qi et al., 2015). However, previous reports of the freeze–thaw method have shown to promote aggregation and instability in the protein. Therefore, it was interesting to investigate the reproducibility, efficiency, and mechanism of the freeze–thaw-based solubilization method for inclusion body proteins. A tough IB protein like hGH and a soft IB protein like L-asparaginase were taken into consideration during this study.

Bioactive protein was recovered from hGH IBs using freeze–thaw as a solubilization method. The purified hGH IBs were solubilized with the freeze–thaw-based method that demonstrated appreciable solubilization efficiency. However, low refolding efficiency, the presence of dimeric species in size exclusion chromatogram, and the low bioactivity of hGH protein insinuated the presence of soluble aggregates and/or misfolded and/or unstable protein species in the refolded and purified hGH proteins. Biophysical studies using far-UV circular dichroism and fluorescence spectroscopy of hGH solubilized in freeze–thaw-based buffer revealed the presence of secondary and tertiary structures in the solubilized state of hGH. This signifies the tendency of the freeze–thaw buffer to protect the secondary and tertiary structures of protein for tough inclusion body protein like hGH and, thus, can be termed as mild denaturing buffer for tough IB protein like hGH. Biochemical studies using HPLC and SDS-PAGE confirmed the presence of soluble aggregates in refolded hGH. Interestingly, the tendency to form soluble aggregates was reported to be higher when freeze–thaw solubilization was performed without a reducing agent such as DTT. This may be because of mismatched disulfide bonded protein species in (IBs), and the addition of DTT would have disrupted all the disulfide bonds and restored a few correct disulfide bonds during refolding. Moreover, it was also demonstrated that the addition of DTT in the freeze–thaw buffer improved its solubilization efficiency. Hence, the addition of DTT while solubilizing a tough IB like hGH could improve solubilization yield as well as the quality of the refolded protein. A high concentration of refolded hGH increased molecular crowding, thereby greater chances of intermolecular interactions between the partially folded hGH species, and thus, it resulted in the formation of soluble aggregates (Fink, 1998). Apart from mismatched disulfide bonds during refolding, the formation of soluble aggregates in the refolded protein species is also dependent on the refolding concentration and incubation period (Goldberg et al., 1991). Interestingly, variation in the refolding concentration (0.2–2 mg/ml) of hGH protein has demonstrated no significant effect on the yield of protein aggregates and refolded protein. This might be attributed to the fact that the refolding concentration of hGH is beyond its conventionally considered critical concentration (0.1–0.2 mg/ml), thereby attaining a saturation in aggregation, and hence, no visible changes in aggregation or refolding yield were observed (Ellis and Minton, 2006; Ciryam et al., 2013, 2015). This suggests that higher than 0.2 mg/ml of refolded hGH leads to a concentration-independent protein aggregation. Surprisingly, the overnight incubation of refolded hGH protein has demonstrated to reduce the soluble aggregates species and, consequently, improve the refolding yield. It is believed that increased duration of refolding period would have given the partially folded intermediates more time to fold properly. It will be interesting to note the effect of increasing the time duration up to a few days on the formation of soluble aggregates during refolding. Overall, avoiding the freeze–thaw-based solubilization, the addition of DTT during solubilization, decreasing the refolding concentration, and overnight incubation of refolded hGH protein reduce the tendency to form soluble aggregates during refolding and improve the overall yield. Soluble aggregates upon long-term storage often cross the energy barrier to nucleation, leading to the formation of insoluble aggregates. This drastically reduces the shelf life of biopharmaceuticals like hGH and often discourages industries for its commercial applications (Berrill et al., 2011). Therefore, instead of the freeze–thaw-based method, several other mild solubilization methods reported might also be used to improve the recovery of bioactive protein from hGH IBs (Table 7).

**TABLE 7:** Percentage yield of each processing step for recovering bioactive hGH protein from IBs using different solubilization buffers.

S. no.	Solubilization buffer	Solubilization yield (%)	Refolding yield (%)	Overall recovery (%)	References
1	30% TFE + 3 M urea + 50 mM Tris	91	85	45	Upadhyay et al., 2016
2	6 M *n*-propanol + 2 M urea + 50 mM Tris	80	76	40	Singh et al., 2012
3	2 M urea + 50 mM Tris, pH 12.5	94	Not determined	50	Patra et al., 2000
4	8 M urea + 50 mM Tris	Not determined	50	21	Singh et al., 2008, 2012
5	6 M β-ME + 2 M urea + 50 mM Tris	97	Not determined	50	Panda et al., 2007
6	2 M urea + 1 mM DTT + 50 mM Tris, freeze–thaw	100	33	14	This study

Similarly, the enzymatically active L-asparaginase protein was recovered from bacterial IBs using freeze–thaw as a solubilization method. The purified L-asparaginase IBs were solubilized with the freeze–thaw-based method that also demonstrated appreciable solubilization efficiency. However, low refolding efficiency, the presence of partially unfolded protein species, and low enzyme activity of L-asparaginase as compared with commercial L-asparaginase insinuated the presence of soluble aggregates and/or misfolded and/or unstable protein species in the refolded and purified L-asparaginase proteins. Biophysical analysis revealed the disruption of the secondary and tertiary structures in the solubilized state of L-asparaginase. This signifies that the freeze–thaw method acts as a strong denaturing buffer for soft inclusion body protein like L-asparaginase and can disrupt the hydrophobic interactions. Biochemical analysis demonstrates a tiny fraction of tetrameric protein and a major fraction of monomeric or dimeric protein species in refolded L-asparaginase. This explains the presence of partially unfolded protein species observed in the fluorescence spectra with purified and solubilized L-asparaginase in comparison with commercial L-asparaginase. It is well known that monomeric subunits of L-asparaginase are bound together with hydrophobic interactions and only tetrameric L-asparaginase is enzymatically active (Verma et al., 2014). The freeze–thaw-rendered disruption of hydrophobic interactions among the monomeric subunits would have destabilized partially the unfolded tetrameric L-asparaginase, thereby possessing low enzymatic activity as compared with commercial L-asparaginase. In contrast, the chaotrope-based method has demonstrated a stable and active tetrameric L-asparaginase at a laboratory scale (Upadhyay et al., 2014; Table 8). At an industrial scale, PEGylation of L-asparaginase has been reported to increase the stability of protein (Meneguetti et al., 2019). These previously reported strategies can offer an alternative to the freeze–thaw-based method for soft IBs and tetrameric protein like L-asparaginase.

**TABLE 8:** Percentage yield of each processing step for recovering enzymatically active L-asparaginase protein from IBs using different solubilization buffers.

S. no.	Solubilization buffer	Solubilization yield (%)	Refolding yield (%)	Overall recovery (%)	References
1	4 M urea + 1 mM PMSF + 20 mM β-mercaptoethanol + 10 mM NaCl + 50 mM Tris	93.2	88.2	50.8	Upadhyay et al., 2014
2	2 M urea + 1 mM DTT + 50 mM Tris, and freeze–thaw	96.6	51.8	25	This study

Interestingly, the freeze–thaw method demonstrated a tendency to form soluble aggregates in case of tough IB protein like hGH, whereas the method destabilized the soft IB like L-asparaginase. Therefore, the rationale for reduced active protein species recovered from bacterial IBs could vary with respect to the intrinsic properties of an inclusion body protein. The selective behavior of the freeze–thaw method in terms of preserving the secondary structure also keeps this method uncategorized. In either of the case, the freeze–thaw-based solubilization method demonstrates the least percentage recovery of active protein species from bacterial (IBs), mainly due to freeze–thaw-rendered protein aggregation or destabilization during refolding. In contrast, slow freezing with quick thawing in the presence of a low concentration of surfactant has recently shown to be a better strategy to avoid protein aggregation (Padhiar et al., 2018). Another approach to improve refolding efficiency could be the addition of chemical additives ensuring no interference in downstream processing. The practicality of the freeze–thaw-based method at the academic and industrial scale will heavily rely on the modifications in the solubilization and refolding strategy that could increase the number of biologically active population of protein species without disturbing its existing solubilization efficiency.

## Data Availability Statement

The raw data supporting the conclusions of this article will be made available by the authors, without undue reservation.

## Author Contributions

PS and AP designed the experiments and prepared the manuscript. PS, JV, NP, TW, ASi, AC, ASa, and MQ performed the experiments. DS proofread the manuscript. All authors read and approved the final manuscript.

## Conflict of Interest

The authors declare that the research was conducted in the absence of any commercial or financial relationships that could be construed as a potential conflict of interest.
